# The Role of Three-Dimensional Scaffolds in Treating Long Bone Defects: Evidence from Preclinical and Clinical Literature—A Systematic Review

**DOI:** 10.1155/2017/8074178

**Published:** 2017-08-09

**Authors:** Alice Roffi, Gopal Shankar Krishnakumar, Natalia Gostynska, Elizaveta Kon, Christian Candrian, Giuseppe Filardo

**Affiliations:** ^1^Nanobiotechnology Laboratory, Rizzoli Orthopaedic Institute, Via di Barbiano 1/10, Bologna, Italy; ^2^Department of Biotechnology, Bannari Amman Institute of Technology, Sathyamangalam, Erode, Tamil Nadu, India; ^3^Department of Biomedical Sciences, Humanitas University, Via Manzoni 113, Rozzano, Milan, Italy; ^4^Humanitas Clinical and Research Center, Via Manzoni 56, Rozzano, Milan, Italy; ^5^Division of Traumatology and Orthopaedics, Regional Hospital of Lugano, Lugano, Switzerland

## Abstract

Long bone defects represent a clinical challenge. Bone tissue engineering (BTE) has been developed to overcome problems associated with conventional methods. The aim of this study was to assess the BTE strategies available in preclinical and clinical settings and the current evidence supporting this approach. A systematic literature screening was performed on PubMed database, searching for both preclinical (only on large animals) and clinical studies. The following string was used: “(Scaffold OR Implant) AND (Long bone defect OR segmental bone defect OR large bone defect OR bone loss defect).” The search retrieved a total of 1573 articles: 51 preclinical and 4 clinical studies were included. The great amount of preclinical papers published over the past few years showed promising findings in terms of radiological and histological evidence. Unfortunately, this in vivo situation is not reflected by a corresponding clinical impact, with few published papers, highly heterogeneous and with small patient populations. Several aspects should be further investigated to translate positive preclinical findings into clinical protocols: the identification of the best biomaterial, with both biological and biomechanical suitable properties, and the selection of the best choice between cells, GFs, or their combination through standardized models to be validated by randomized trials.

## 1. Introduction

Traumatic long bone defects still represent a clinical challenge for orthopaedic surgeons. In fact, a critical size defect requires invasive surgical procedures to reconstitute the structural integrity of the collapsed bone. This does not provide fully satisfactory results entailing a significant socioeconomic burden [1, 2]. Despite all recent innovations in bone repair techniques, autologous bone grafting (ABG) is still considered the “gold standard” treatment for long bone defects. However, ABG presents major limitations due to related drawbacks such as longer operating time, little availability of material, and significant morbidity [3–8]. Other options could be the treatment with allografts or xenografts, but some disadvantages have also been reported for these methods, such as immune rejection, slow and only partial integration, absorption and substitution with new bone, graft sequestration, and failures [9].

The concept of bone tissue engineering (BTE) has been developed to overcome problems associated with conventional methods. The typical paradigm of BTE is constituted by the four biological prerequisites which include osteogenic cells, osteoinductive stimulus, osteoconductive matrix scaffolds, and mechanical environment (the diamond concept) [10]. These promote signalling cascades such as osteogenesis, chondrogenesis, and angiogenesis in an orchestrated spatiotemporal manner, leading to bone regeneration [10]. In this context, it is crucial for the scaffold to have a proper macroporous structure, good degradability, and osteoconductive properties [11–13]. Thus, three-dimensional (3D) scaffolds have been developed with hierarchically organized structures similar to healthy bone and with the ability to yield well-organized bone regeneration [14, 15]. Moreover, the important role of growth factors (GFs) in bone remodelling and osteogenesis, by accelerating chemotaxis, proliferation, and differentiation of bone cells, has been largely described [16–18]. In this light, it has become common to add augmentation strategies such as osteoinductive factors like bone morphogenetic proteins (BMPs), vascular endothelial growth factor (VEGF), basic fibroblast growth factor (bFGF), platelet rich plasma (PRP), and bone marrow derived stem cells (BMSCs) to further stimulate bone healing in critical size defects. However, many questions remain unanswered (choice of scaffold, cell source and concentration, type of GFs, etc.) thereby causing uncertainty on the potential of available technologies as well as on the choice of the most suitable strategy.

The main objective of this systematic review was to assess the BTE strategies available in preclinical and clinical settings, in order to analyse the current evidence supporting the use of this approach for the treatment of long bone defects.

## 2. Methods

**Figure 1:**
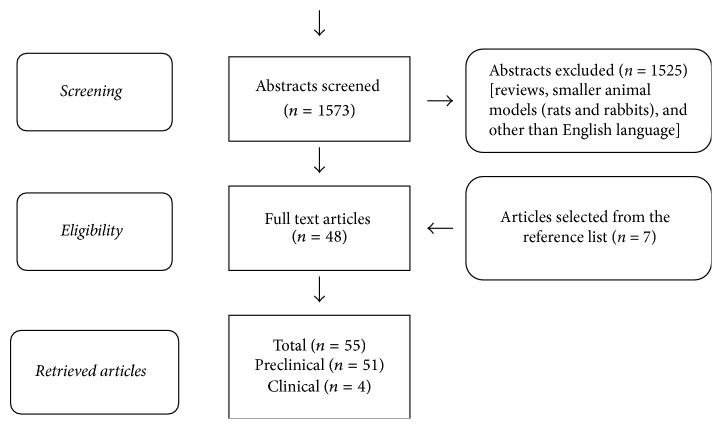
PRISMA flowchart of the paper's selection process.

A systematic literature screening was performed by two independent reviewers (GS and NG) on the PubMed database, searching for both preclinical and clinical studies on 3D synthetic scaffolds with organized structures for long bone defects developed to treat defects of the upper/lower extremities. In particular, the research criteria included studies published in English language until February 2017. The following string was used: “(Scaffold OR Implant) AND (Long bone defect OR segmental bone defect OR large bone defect OR bone loss defect)” (Figure 1). Among preclinical publications, only studies on large animal models were selected. After an initial screening of all abstracts, selected full texts were analysed and separated into preclinical and clinical studies. Reference lists were also screened to identify further papers. All articles dealing with other types of bone defects not involving upper/lower extremities or studies without scaffolds were excluded. Moreover, biomaterials in form of granules, sponges, or powders were excluded and studies with only autografts or allografts were also excluded.

## 3. Results

The PubMed search analysis retrieved a total of 1573 articles and, following the inclusion criteria, 51 preclinical studies [19–69] and 4 clinical studies [70–73] were identified and included in the present analysis. Details of preclinical studies are reported in Table 1 (scaffold alone) and Table 2 (augmented scaffold) and clinical papers in Table 3.

### 3.1. Preclinical Studies

**Figure 2:**
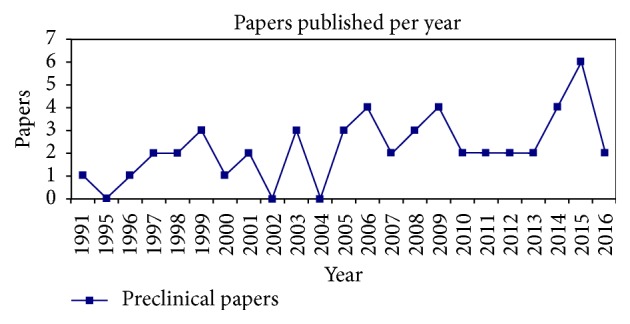
Preclinical studies published over time.

In the past, few years there has been a progressive increase in the number of publications for scaffold treatments in the preclinical field, as shown in Figure 2. The most commonly investigated large animal model was sheep 30/51, followed by dog 13/51, goat 6/51, and monkey 2/51. A composite scaffold derived from a combination of different biomaterials was the most common investigated choice 33/51, followed by coral scaffolds 7/51, hydroxyapatite (HA) 5/51, tricalcium phosphates (TCP) 4/51, alumina derived scaffolds 1/51, and bioactive glass 1/51. Out of 51 articles, 12 reported the use of the scaffold alone and 39 supplemented the scaffold with an augmentation strategy.

Concerning the scaffolds alone application (Table 1), only 1/12 articles compared the effects of scaffold with ABG treatment and reported no significant differences in the final outcome. The remaining 11/12 articles reported good results, with evidence of enhanced new bone formation and remodelling with functional recovery and segmental defect healing. The other 39/51 articles reported the use of scaffolds with cells, GFs, or their combination (Table 2).

The use of cells was described in 22/39 papers; 2/22 of which used bone marrow concentrates (BMC), 19/22 expanded bone marrow MSCs (BMSCs), and 1/22 expanded adipose tissue MSCs (ADMSCs). Overall results showed that the use of cells (both autologous or allogenic) in combination with scaffolds had an additional positive effect compared to the scaffold alone, with the ability to heal segmental defects with improved bone regeneration, in terms of radiological and histological results.

The use of scaffolds with GFs augmentation was reported in 8/39 papers. Out of these, 7/8 papers reported the use of BMPs (freshly extracted BMPs from bone in 4/7, BMP-2 in 1/7, and BMP-7 in 2/7) and 1/8 paper reported the use of rhTGF*β*-3. Results of 5/8 articles reported positive effects, mainly emphasizing that GFs largely assisted the healing process of critical sized defects due to their osteoinductive properties. On the other hand, 2/8 papers, 1 on freshly extracted BMPs from bone and 1 on rhTGF*β*-3, reported inferior and similar results, respectively, when compared to ABG treatment.

Four papers out of 39 compared the use of different cell sources, or cells versus GFs. Among these, 2 papers reported superior results for BMSCs when compared to PRP or BMC augmentation. One paper showed no significant differences between BMC and BMP-7 added to a HA scaffold, with better results compared to the scaffold alone and similar results compared to ABG. Interestingly, one paper comparing the effects of PRP, mesenchymal progenitor cells (MPCs), orofacial skeleton osteoblasts (mOBs), and axial skeleton osteoblasts (tOBs) found that the MPCs group produced better results when compared to other biological enhancers.

The use of cells and GFs in combinations was reported in 5/39 papers with different study designs, which prevents us drawing an overall conclusion. The combination of scaffolds, cells, and GFs (either PRP or AdBMP-7) provided superior results compared to the scaffold/cell construct in 2/5 papers. On the contrary, 2/5 studies showed worse results for scaffold/BMSCs/PRP compared to a scaffold/BMP-7 construct. Finally, one study showed superior results combining 2 cell sources, such as OBs (osteoblasts cells) and ECs (endothelial cells) compared to the use of a single cell type (ECs).

### 3.2. Clinical Studies

The literature search identified 4 clinical papers that met the inclusion criteria. Two of the 4 clinical trials used scaffolds without any augmentation and the other 2 reported a cell augmentation approach. An HA scaffold was used in 3/4 papers followed by beta-TCP in 1 study.

In 2000 Werber et al. [70] presented a study about the treatment of distal radius fractures with HA ceramic from processed bovine spongiosa. The scaffold was implanted in 14 patients followed up for up to 15 months after surgery. Magnetic resonance imaging (MRI) scans showed integration of the biomaterial with the surrounding tissue and bone regeneration in 13 patients, without any adverse events (MRI was nondiagnostic in 1 case where a broken screw caused extensive artifacts). However, only in 1 patient complete radius regeneration was documented. One year later, a case series performed by Quarto et al. in 2001 [71], followed up by Marcacci et al. in 2007 [73], described the treatment of tibia, humerus, and ulna segmental defects with porous HA ceramic scaffold seeded with BMSCs, expanded by culture with fetal calf serum and FGF-2, and suspended in fibrin glue activated with thrombin to form the final ceramic-cell composite. Radiographic and computed tomography (CT) analyses reported complete integration between the scaffold and host bone starting from 5 to 7 months after surgery in all 4 patients. In 3 of them, whose evaluations were available at longer follow-up times, this trend was confirmed until 6 to 7 years after surgery. Additionally, no major complications were reported in the early or late periods after surgery. In 2005, Arai et al. [72] investigated the use of beta-TCP scaffold for the treatment of 14 patients who had fibula resections to be used as autogenous bone grafts. These were used for the reconstruction of large segmental defects in benign bone tumours of the extremities and pseudarthrosis of the tibia. At an average time of 9.3 months after surgery, scaffold absorption and new bone formation were observed. However, according to the radiographic evaluation, complete regeneration of the fibula occurred only in one case. In 2 paediatric patients, implant replacement by neotissue was noticed already at 3.2 months after surgery.

## 4. Discussion

In this systematic review, the interest on the scaffold based strategy to treat long bone defects was documented by the great amount of preclinical papers (even though highly heterogenous) published over the past few years, showing overall promising findings in terms of radiological and histological evidence, with the ability to treat segmental defects in large animal models. Unfortunately, this in vivo situation is not reflected by a corresponding clinical impact of this treatment approach, with few published clinical papers, highly heterogeneous and presenting small patient populations.

In orthopaedic surgery, critical size bone defects derived from nonunion, trauma, or tumours are a challenging problem, from both a social and economic perspective. In fact, in Europe, the total cost of treatment of nonunion defects is between 10000 and 100000 € per patient, with an amount of around 1 million bone operations every year [74]. Bone grafting as an ABG still represents a gold standard for regenerating bone defects. Approximately, 2.2 million bone grafting procedures are performed worldwide every year and the majority involve ABG followed by allograft [74]. Although ABG has been widely used for the treatment of long bone defects with a high success rate reported to range between 70 and 95%, a 50% failure rate has also been reported [74, 75]. These failures of autologous grafting procedures can be related to morbidity, pain, and many other perioperative and postoperative complications caused by the harvesting process. Allograft can be a good substitute of ABG by avoiding donor-site morbidity and pain, but immunorejection, bacterial infections, and viral transmission are limitations of this procedure [76, 77], which still offers not optimal outcomes. In fact, the internal repair (revascularization and substitution of the original graft bone with new host bone) progresses slowly and seems to be confined only to the superficial surface and the ends of the graft [78, 79]. Furthermore, the rate of complications increases proportionally to the size of the defect that have to be replaced [79], and among these, allograft fractures are the major drawback [80]. Moreover, the cost of allo- or autografting can be high, which further prompts the development of other strategies such as BTE [74].

In the last 20 years, the number and variety of biomaterials developed for the treatment of segmental bone defects have been increasing, especially in preclinical setting. This review was focused on solid biomaterials, with 3D scaffolds that can mimic bone structure and composition when implanted in vivo into the defects, with results documented in studies on large animals (to better reproduce human conditions) and in clinical settings.

HA and ceramic calcium phosphates, such as TCP that resemble mineral components of bone, are the most used materials in both preclinical and clinical settings, offering a biological response similar to that of natural bone [81]. This ability is probably due to their suitable chemical composition, porosity, and mechanical properties [81], which may differ among scaffolds. TCP ceramics possess sufficient porosity, which may be adjusted to favour neotissue in-growth; however, their biomechanical resistance is limited compared to HA [76, 81]. On the other hand, a key aspect that could affect the final clinical outcome is the degradation time of the scaffold: in this light, the mechanical properties of HA are counterbalanced by its slow degradation by osteoclasts, which is approximately 2–5 years, while a faster biodegradation, as in the case of TCP that is degraded in 1 year, could led to a faster loss of mechanical strength [81]. Indeed, slow biodegradation of the HA scaffold was observed in the clinical trial of Werber et al. where at the 15-month follow-up porous HA was not completely resorbed and replaced by new bone [70]. Moreover, in the clinical study performed by Marcacci et al., HA ceramics were not absorbed even after 7 years [73]. On the contrary, Arai et al. used a degradable beta-TCP scaffold and observed its absorption and deposition by new bone in 12 out of 14 patients 9 months after surgery, although its regeneration was mainly incomplete, with only one adult patient presenting a complete regenerated fibula [72]. Therefore, the design of a mechanically stable material, suitable for load-bearing in segmental defects, which is also bioabsorbable, remains challenging [74–77].

Another interesting aspect in the field of biomaterials is related to the diamond concept, which involves a combined approach by combining osteoinductive factors (cells or GFs) with 3D scaffolds and was the most investigated option in preclinical settings. Due to the heterogeneity of these studies (different cell sources or GFs used, application protocol, dosage,  …), it is difficult to draw a conclusion about the best augmentation procedure able to enhance bone healing. In fact, overall positive results have been reported, but only few papers compared the different augmentation strategies in the preclinical model, thus leaving literature findings inconclusive. Among GFs, the most exploited strategy involves the BMPs family, whose discovery dates back to the end of the nineteenth century and drew an increasing attention in the scientific community, with a large literature including also the overall good results of the clinical application, although documented adverse events and some controversial reported outcomes limited their impact in the clinical practice [82]. While other isolated GFs have been explored as well, the currently most exploited strategy to deliver GFs is the use of blood derivatives such as platelet concentrates [5]. PRPs are proposed as powerful tools for tissue healing, thanks to the many GFs contained in their alpha granules, which can be delivered concentrated but in physiologic proportions [5]. The evidence for PRP osteogenic potential has been suggested by several in vitro studies. PRP addition in culture medium promoted the proliferation and differentiation of MSCs, PRP can improve cell chemokinesis and chemotaxis through cytoskeleton reorganization and accelerate cell migration, thus influencing cells mobility, and antimicrobial effects have been suggested as well, which are highly desirable in relation to a surgical bone application [5]. Nonetheless, besides the aforementioned beneficial roles, in vitro studies have also shown controversial results on PRP potential to favour bone healing, which remains a debated aspect [5]. Among augmentation strategies, MSCs represent an exciting and promising cell population for bone regeneration, especially when tissue engineering or biomaterials are applied [83]. Their potential of “natural system of tissue repair” has been suggested by studies in different fields of medical application, and they have been extensively investigated also for bone tissue engineering. MSCs have been firstly identified in bone marrow, but nowadays they have been isolated also from other human sources, which are explored in terms of potential applicability in the clinical practice. In this light, considering that cell amplification by culture is not free from the dangers of bacterial contamination and entails economic and regulatory limitations, the use of concentrates is gaining increasing interest, despite the lower number of cells with respect to cell expansion process [83]. While autologous cells have been preferred up to now in the clinical scenario, the possibility of simplifying the procedure by taking advantage of allogeneic cells seems attractive and is currently explored in terms of potential and risks as well. This preclinical review documented many studies applying allogenic cells, but only one study directly compared autologous and allogenic sources, showing overall similarly good results [47]. Finally, in light of future advancement of the augmentation strategies potential, gene therapy is investigated to improve the repair of tissues by providing a temporarily and spatially defined expression of therapeutic genes at the site of injury [84]. In fact, adapting tissue engineering platforms to gene transfer approaches mediated by viral vectors is an attractive tool to circumvent both the limitations of the current therapeutic options to promote an effective healing of the tissue. Several gene transfer vehicles have been developed to modify human cells and tissues from musculoskeletal system, and future studies should demonstrate whether this technology might provide an effective solution compared to the other available augmentation strategies for bone healing [84]. Therefore, while cells and GFs are highly attractive for the healing of segmental defects, the identification of the best application strategy still requires investigation with specifically designed studies to compare cells from different sources and with different manipulation, GFs, and their combinations.

The clinical scenario does not reflect the high research activity documented by the preclinical literature: only 4 papers were found, all with a small patient number, different study design, and heterogeneous pathologies treated. The search identified 2 clinical trials using scaffolds without any augmentation, while another 2 reported a cell augmented scaffold approach. Among different sources of MSCs, bone marrow was the most commonly used for BTE in orthopaedic surgery [75, 76]. BMSCs can be easily isolated from the iliac crest, immediately injected or implanted with the carrier into the defect or expanded in vitro before implantation. In two clinical studies presented in this review, expanded BMSCs were seeded on a HA porous scaffolds, both showing satisfactory results [71, 73]. Nevertheless, lack of suitable controls did not allow verifying whether the positive clinical outcome is derived from the added regenerative potential of the implanted cells or from the implanted scaffold itself, by promoting the body own regenerative potential. Thus, while there is a huge demand to increase the regenerative potential of scaffolds, the difficulties in translating preclinical findings into clinical practice leave many questions still unanswered [77]. Further studies are needed to develop strategies with scaffold, cells, and GFs combined to overcome the results of autografts and offer a suitable treatment option to rapidly regenerate bone segmental defects.

## 5. Conclusion

This systematic research of the literature documented a growing interest in scaffold based approaches applied in preclinical settings to promote tissue regeneration in long bone defects of critical size. However, this evidence did not translate into a similar interest in the clinical scenario, characterized by only 4 papers published, with low quality and heterogeneous study designs. Several interesting aspects have been underlined by preclinical literature, in particular with regard to the benefit of an augmentation strategy to enhance the regenerative potential of the biomaterial. These should be further investigated in order to translate positive preclinical findings into clinical protocols: first of all, to identify the best biomaterial for long bone defects, with both biological and biomechanical suitable properties, and then to select the best choice between cells, GFs, or their combination, in order to provide the best treatment option for patients affected by long bone defects.

## Figures and Tables

**Table 1:** Complete details of 12 preclinical papers identified in this systematic review focusing on the usefulness of scaffolds alone in treating long bone defects.

Authors	Biomaterials	Animal model	Results	Effects
Boyde et al., 1999 (Bone) [19]	(1) HA	Sheep tibial defect (3.5 cm)	SEM: + BSE: +	+
Marcacci et al., 1999 (Cal Tiss Int) [20]	(1) HA	Sheep tibial defect (3.5 cm)	X-ray: + Hist: +	+
Zhang et al., 2001 (J Biomat Mat Res) [21]	(1) HA-TCP	Dog femoral defect (1.5 cm)	Mech: +	+
Mastrogiacomo et al., 2006 (J Tiss Eng) [22]	(1) Si-TCP	Sheep tibial defect (4.8 cm)	X-ray: + Hist: + *µ*CT: +	+
Sarsilmaz et al., 2007 (Acta of Bioeng & Biomech) [23]	(1) HA-PE	Dog radial defect (1.5 cm)	X-ray: + Hist: + SEM: +	+
Schneiders et al., 2009 (J Orth Res) [24]	(1) HA-COL (2) HA-COL-CS	Sheep tibial defect (3 cm)	X-ray: + Hist: + *µ*CT +	+ (for HA-COL-CS)
Nandi et al., 2009 (Res Vet Sci) [25]	(1) Untreated (2) Bioactive glass	Sheep radial defect (1.2 cm)	X-ray: + Hist: +	+
Nair et al., 2010 (J Tiss Eng Pt A) [26]	(1) HASi	Goat femoral defect (2 cm)	X-ray: + Hist: + *µ*CT: +	+
Reichert et al., 2011 (Int Ortho) [27]	(1) Untreated (2) mPCL-TCP (3) PLDLLA-TCP-PCL (4) ABG	Sheep tibial defect (2 cm)	X-ray: + Hist: + *µ*CT: + Mech: +	+ (similar results of ABG)
Rentsch et al., 2012 (Biomatter) [28]	(1) PCL-Coll I-CS	Sheep tibial defect (3 cm)	X-ray: + Hist: + *µ*CT: + Mec: +	+
Kim et al., 2015 (Biomed Res Inter) [29]	(1) HA/alumina (2) HA/alumina-medullary canal (3 mm)	Dog tibial defect (2 cm)	X-ray: + *µ*CT: + Fluorescent labelling: +	+ (for HA/alumina-medullary canal)
Li et al., 2016 (Biomed Mat) [30]	(1) Baghdadite (2) Baghdadite-PCL-nBG	Sheep tibial defect (3 cm)	X-ray: = Hist: = *µ*CT: = Mech: +	+ (for Baghdadite-PCL-nBG)

HA: hydroxyapatite, HA-TCP: hydroxyapatite-tricalcium phosphate, Si-TCP: silicon stabilized tricalcium phosphate, HA-PE: hydroxyapatite-polyethylene, HA-COL: hydroxyapatite-collagen, HA-COL-CS: hydroxyapatite-collagen-chondroitin sulphate, HASi: calcium silicate, tricalcium phosphate, and hydroxyapatite, mPCL-TCP: medical grade polycaprolactone-tricalcium phosphate, (PLDLLA)- TCP-PCL: poly(L-lactide-co-D,L-lactide)- polycaprolactone-tricalcium phosphate, ABG: autologous bone graft, PCL-Coll I-CS: polycaprolactone-collagen- chondroitin sulphate, HA-alumina: hydroxyapatite-alumina, Baghdadite: Ca_3_ZrSi_2_O_9_, Baghdadite-PCL-nBG: Ca_3_ZrSi_2_O_9_-polycaprolactone-bioactive glass nanoparticles, SEM: scanning electron microscopy, BSE: backscattered electron imaging, Hist: histological analysis, *µ*CT: microcomputed tomography, Mech: mechanical analysis, X-ray: radiological analysis, +: positive effects, −: negative effects, and = : no significant difference.

**Table 2:** Complete details of 39 preclinical papers identified in this systematic review focusing on the usefulness of scaffolds with augmentation in treating long bone defects.

	Authors	Biomaterials	Animal model	Cells/Gfs type and dose	Analysis	Scaffold results
Cells	Grundel et al., 1999 (Clin. Orthop. Relat. Res) [31]	(1) Untreated (2) HA-TCP-Granular-BMCs (3) HA-TCP-Block-BMC (4) ABG	Dog ulna defect (2.5 cm)	BMCs	X-ray: + Hist: + Mech: +	+ (For HA-TCP-Block BMC)
	Johnson et al., 1996 (J. Orth. Res) [32]	(1) TCP (2) TCP-BMCs (3) HA-COL (4) HA-COL-BMCs (5) ABG	Dog radial defect (2.5 cm)	BMCs	X-ray: + Hist: + Mech: +	+ (For TCP-BMCs)
	Bruder et al., 1998 (J. Bone & Joint Surg) [33]	(1) Untreated (2) HA-TCP (3) HA-TCP-BMSCs	Dog femoral defect (2.1 cm)	BMSCs (7.5 × 10^6^/ml)	X-ray: + Hist: +	+
	Kon et al., 1999 (J. Biomed. Mat. Res) [34]	(1) HA (2) HA-BMSCs	Sheep tibial defect (3.5 cm)	BMSCs (2.5 × 10^5^/ml)	X-ray: + Hist: + SEM: + Mech: +	+
	Arinzeh et al., 2003 (J. Bone & Joint Surg) [35]	(1) Untreated (2) HA-TCP (3) HA-TCP-allogenic BMSCs	Dog femoral defect (2.1 cm)	BMSCs (7.5 × 10^6^/ml)	X-ray: + Hist: +	+
	Bensaïd et al., 2005 (J. Tiss. Eng. A) [36]	(1) Untreated (2) CHA (3) CHA-BMSCs (4) ABG	Sheep metatarsus defect (2.5 cm)	BMSCs (1 × 10^7^/ml)	X-ray: + Hist: +	+
	Mastrogiacomo et al., 2005 (Orthod. Craniofac. Res) [37]	(1) HA-TCP-BMSCs	Sheep tibial defect (5 cm)	BMSCs (0.5–1.0 × 10^8^/ml)	X-ray: + Hist: + *µ*CT: +	+
	Viateau et al., 2006 (J. Orth. Res) [38]	(1) Untreated (2) Coral (3) Coral-BMSCs	Sheep metatarsus defect (2.5 cm)	BMSCs (8.28 ± 1.32 × 10^6^/implant)	X-ray: + Hist: +	+
	Zhu et al., 2006 (J. Tiss. Eng) [39]	(1) Coral (2) Coral-BMSCs	Goat femoral defect (2.5 cm)	BMSCs (20 × 10^6^/ml)	X-ray: + Hist: + Mech: +	+
	Mastrogiacomo et al., 2007 (J. Biomat) [40]	(1) Si-TCP (2) Si-TCP-BMSCs	Sheep tibial defect (4 cm)	BMSCs (0.5–1.0 × 10^8^/ml)	Micro-diffraction: + *µ*CT: +	+
	Liu et al., 2008 (J. Mat Sci: Mat Med) [41]	(1) Untreated (2) *β*-TCP (3) *β*-TCP-BMSCs	Goat tibia defect (2.6 cm)	BMSCs (2 × 10^7^/ml)	X-ray: + Hist: + *µ*CT: + SEM: +	+
	Giannoni et al., 2008 (J. Tiss. Eng. Regen. Med) [42]	(1) ABG (2) HA-Si-TCP (3) HA-Si-TCP-BMSCs	Sheep tibial defect (4.5 cm)	BMSCs (70–100 × 10^6^)	X-ray: + Hist: +	+
	Nair et al., 2008 (J. Biomed. Mater. Res. A) [43]	(1) HASi (2) HASi + BMSCs	Goat femoral defect (2 cm)	BMSCs (1 × 10^5^/cm^2^)	X-ray: + Hist: + *µ*CT: + SEM: +	+
	Niemeyer et al., 2010 (J. Tiss. Eng. A) [44]	(1) Untreated (2) HA-COL-BMSCs (allogenic) (3) HA-COL-BMSCs (xenogenic)	Sheep tibial defect (3 cm)	BMSCs (2 × 10^7^/ml)	X-ray: + Hist: +	+ (For allogenic BMSCs)
	Cai et al., 2011 (J. Biomat) [45]	(1) CHA (2) CHA-BMSCs (vascularized) (3) CHA (vascularized) (4) CHA-BMSCs	Dog fibula defect (1 cm)	BMSCs (20 × 10^6^/ml)	Hist: + *µ*CT: + Hist: +	+ (For CHA-BMSCs vascularized)
	Manassero et al., 2013 (J. Tiss. Eng. A) [46]	(1) Coral (2) Coral-BMSCs	Sheep metatarsus defect (2.5 cm)	BMSCs (7.5 ± 1.2 × 10^6^/implant)	X-ray: + Hist: + *µ*CT: +	+
	Berner et al., 2013 (Acta. Biomater) [47]	(1) mPCL-TCP (2) mPCL-TCP-BMSCs (Autologous) (3) mPCL-TCP-BMSCs (allogenic) (4) ABG	Sheep tibial defect (3 cm)	BMSCs (35 × 10^6^/500 *µ*l)	X-ray: + Hist: + *µ*CT: = Mech: =	+ (Similarly for both autologous and allogenic cells)
	Fan et al., 2014 (J. Biomat) [48]	(1) Untreated (2) TCP-*β* (3) TCP-*β*-BMSCs (4) TCP-*β*-BMSCs-saphenous vascular (5) TCP-*β*-BMSCs-fascia flap	Monkey tibial defect (2 cm)	BMSCs (5 × 10^6^/implant)	X-ray: + Hist: + SPECT: + MRI: +	+ (For TCP-*β*-BMSCs-saphenous vascular)
	Yoon et al., 2015 (J Vet Sci) [49]	(1) Untreated (2) ASA (3) ASA-AdMSCs (4) ASA-*β*-TCP (5) ASA-*β*-TCP-AdMSCs	Dog ulna defect (1.5 cm)	ADMSCs (1 × 10^6^/50 *µ*l)	X-ray: + Hist: +	+ (For ASA-Ad-MSCs)
	Berner et al., 2015 (Stem cells Trans Med) [50]	(1) PCL-HA (2) PCL-HA-allogenic BMSCs (3) ABG	Sheep tibial defect (3 cm)	BMSCs (100 × 10^6^)	X-ray: + Hist: + *µ*CT: + SEM: + Mech: +	+
	Masaoka et al., 2016 (The Open Biomed Eng J) [51]	(1) *β*-TCP (2) *β*-TCP-BMSCs	Monkey femur defect (5 cm)	BMSCs (1.3–4.1 × 10^6^/ml)	X-ray: + Hist: +	+
	Smith et al., 2017 (J Tiss Eng Reg Med) [52]	(1) Untreated (2) PLLA-PCL (3) PLLA-PCL-BMSCc	Sheep tibial defect (3.5 cm)	BMSCs (1 × 10^7^/implant)	Hist: + *µ*CT: + Mech: +	+

GFs	Kirker-Head et al., 1995 (Clin. Orthop. Relat. Res) [53]	(1) 2 mg BMP-2-PLGA-blood (2) 4 mg BMP-2-PLGA-blood (3) PLGA-blood	Sheep femoral defect (2.5 cm)	BMP-2 (2 mg and 4 mg)	X-ray: + Hist: +	+ (For both concentrations of BMP-2
	Sciadini et al., 1997 (J. Orth. Res) [54]	(1) Coral (2) Coral-BMP (3) ABG	Dog radial defect (2.5 cm)	BMP extract (3 mg/implant)	X-ray: + Hist: + Mech: +	+
	Gao et al., 1997 (Int. Ortho) [55]	(1) Coral (2) Coral-BMP	Sheep tibial defect (1.6 cm)	BMP extract (100 mg/implant)	X-ray: + Hist: + Mech: +	++)
	Tuominen et al., 2001 (Ann Chir Gynaecol) [56]	(1) HA (2) HA-BMP (3) ABG	Dog ulna defect (2 cm)	BMP extract	X-ray: − Hist: − Mech: −	− (With or without BMPs but inferior to ABG)
	Hu et al., 2003 (J. Biomed. Mater. Res. A) [57]	(1) Untreated (2) HA-COL-PLA (3) HA-COL-PLA-BMP	Dog radial defect (2 cm)	BMP extract (30 mg/implant)	X-ray: + Hist: + DEXA: +	+
	Cook et al., 2005 (J. Biomed. Mat. Res) [58]	(1) 3.5 mg BMP-7 (2) 3.5 mg BMP-7-CMC (3) 1.75 mg BMP-7 (4) 1.75 mg BMP-7-CMC	Dog ulna defect (2.5 cm)	BMP-7 (3.5 mg/implant and 1.75 mg/implant)	X-ray: = Hist: = Mechanical: =	=
	Maissen et al., 2006 (J. Orth. Res) [59]	(1) Untreated (2) PLA (3) PLA-rhTGF*β*-3 (4) ABG	Sheep tibial defect (1.8 cm)	rhTGF*β*-3 (269.4 *µ*g/implant)	*µ*CT: − X-ray: − Mech: −	− (Inferior to ABG)
	Cipitria et al., 2015 (Act Biomat) [60]	(1) mPCL-TCP (2) mPCL-TCP+BMP-7	Sheep tibial defect (3 cm)	BMP-7 (3.5 mg/implant)	Hist: + *µ*CT: + BSE: + SAXS: + Mech: +	

Comparisons	Petite et al., 2000 (Nat Biotech) [61]	(1) Coral-BMSCs (2) Coral-BMCs (3) Coral	Sheep metatarsus defect (2.5 cm)	BMSCs (3.25 ± 0.25 × 10^7^ cells/ml) BMCs (7 × 10^6^ ± 1 × 10^6^)	X-ray: + Hist: +	+ (For BMSCs)
	den Boer et al., 2003 (J Orth Res) [62]	(1) Untreated (2) ABG (3) HA (4) HA-BMP-7 (5) HA-BMCs	Sheep tibial defect (3 cm)	BMP-7 (2.5 mg/implant) BMCs	X-ray: + Hist: = Mech: +	+ (For both HA + BMP-7 and HA + BMCs)
	Filardo et al., 2014 (J Tiss Eng A) [63]	(1) BioSiC(HA-COL) (2) BioSiC(HACOL) + PRP (3) BioSiC(HA-COL) + BMSCs	Sheep metatarsus defect (2 cm)	BMSCs (4 ± 2 × 10^6^/ml)	X-ray: = Hist: +	+ (For BMSCs)
	Berner et al., 2015 (J. Tiss. Eng. Reg. Med) [64]	(1) mPCL-TCP-PRP (2) mPCL-TCP-allogenic-MPC (3) mPCL-TCP-allogenic-mOB (4) mPCL-TCP-allogenic-tOB	Sheep tibial defect (3 cm)	MPCs, mOB, tOB (35 × 10^6^ cells)	X-ray: + Hist: + *µ*CT: + Mech: +	+ (For mPCL-TCP-allogenic MPC)

Combinations	Nair et al., 2009 (Acta. Biomat) [65]	(1) HASi (2) HASi + BMSCs (3) HASi + BMSCs + PRP	Goat femoral defect (2 cm)	BMSCs (1 × 10^5^ cm^2^)	X-ray: + Hist: +	+ (For HASi + BMSCs + PRP)
	Zhu et al., 2009 (J Orth Res) [66]	(1) Coral-BMSCs (2) Coral-AdBMP-7- BMSCs	Goat femoral defect (2.5 cm)	BMSCs (5 × 10^7^/ml)	X-ray: + Hist: + Mech: +	+ (For Coral-AdBMP-7-BMSCs)
	Reichert et al., 2012 (Sci Trans Med) [67]	(1) Untreated (2) mPCL-TCP (3) mPCL-TCP-BMSCs + PRP (4) mPCL-TCP-BMP-7 (5) ABG	Sheep tibial defect (3 cm)	BMSCs (35 × 10^6^ cells/ 250 *µ*l) BMP-7 (3.5 mg/implant)	X-ray: + Hist: + *µ*CT: + Mech: +	+ (For mPCL-TCP-BMP-7)
	Li et al., 2014 (Orthop) [68]	(1) TCP-*β*-OCs-ECs (2) TCP-*β*-ECs	Sheep femoral defect (3 cm)	OCs and ECs (2 × 10^6^/ml)	X-ray: + Hist: +	+ (For TCP-*β* OCs-ECs)
	Ronca et al., 2014 (J Biomat Appl**) **[69]	(1) HYAFF11® (2) HYAFF11 + PRP + BMSCs (3) HYAFF11 + BMP-7	Sheep metatarsus defect (2 cm)	BMSCs (1 × 10^6^/ml) BMP-7 (0.4 *µ*l/ml)	Hist: +	+ (For HYAFF11 + BMP-7)

HA: hydroxyapatite, HA-TCP: hydroxyapatite-tricalcium phosphate, HA-COL: hydroxyapatite-collagen, TCP: tricalcium phosphate, ABG: autologous bone graft, PLGA: poly(D,L-(lactide-co-glycolide)), HA-COL-PLA: hydroxyapatite-collagen-poly(L-lactic acid), CMC: carboxyl methyl cellulose, CHA: coral hydroxyapatite, PLA: poly(L/DL/lactide), Si-TCP: silicon stabilized tricalcium phosphate, *β*-TCP: beta tricalcium phosphate, HA-Si-TCP: hydroxyapatite silicon stabilized tricalcium phosphate, HASi: calcium silicate, tricalcium phosphate, hydroxyapatite, mPCL-TCP: medical grade polycaprolactone-tricalcium phosphate, BioSic(HA-COL): biomorphic silicon carbide hydroxyapatite-collagen, HYAFF11: poly-*ε*-caprolactone-poly-L-lactic acid with hyaluronan derivatives, PLLA-PCL: poly(L-lactic acid)-poly(*ε*-caprolactone), ASA: autologous serum albumin, PCL-HA: polycaprolactone-hydroxyapatite, AdBMP-7: adenovirus mediated bone morphogenetic protein 7, ADMSCs: adipose derived mesenchymal stem cell, BMCs: bone marrow concentrates, MPCs: mesenchymal progenitor cells, tOBs: axial skeleton osteoblasts, mOBs: orofacial skeleton osteoblasts, BMP: bone morphogenetic protein, BMP-2: bone morphogenetic protein-2, BMP-7: bone morphogenetic protein-7, BMSCs: bone marrow derived mesenchymal stem cell, rhTGF-*β*3: recombinant transforming growth factor beta 3, PRP: platelet rich plasma, OCs: osteoblast cells, ECs: endothelial cells, SEM: scanning electron microscopy, SAXS: small angle X-ray scattering, DEXA: dual-energy X-ray absorptiometry, Hist: histological analysis, *µ*CT: microcomputed tomography, Mech: mechanical analysis, X-ray: radiological analysis, +: positive effects, −: negative effects, and =: no significant difference.

**Table 3:** Complete details of 5 clinical papers identified in this systematic review focusing on the usefulness of scaffolds with or without augmentation in treating long bone defects.

References	Study type	Pathology	Scaffold	Augmentation	Number of patients	Follow-up	Results
Werber et al., 2000 (J Hand Surg) [70]	Case series	Distal radius fracture	HA ceramic from bovine spongiosa (Merck Biomaterials)	—	14	15 m	Bone healed around the graft material and fibrovascular ingrowth within the scaffold observed
Quarto et al., 2001 (N Engl J Med) [71]	Case series	Tibia, humerus, and ulna defect	Porous HA ceramic (Finceramica)	BMSCs (2 × 10^7^ cells/mL)	3	15–27 m	Limb function recovered for all patients; good integration with the host bones by the second month after surgery in all cases
Arai et al., 2005 (Clin Orthop Relat Res) [72]	Case series	Fibula resections for use as autograft for reconstruction of large segmental defects of tibia	TCP (Osferion Olympus)	—	14	4–42 m (mean 17 m)	In 12 patients scaffold was absorbed and replaced by newly formed bone at an average 9.3 months after surgery. In all children, new bone formation was at 3.2 months; only one patient had complete regeneration of the fibula
Marcacci et al., 2007 (Tissue Engineering) [73]	Case series	Tibia, humerus, and ulna defect	Porous HA ceramic (Finceramica)	BMSCs (2 × 10^7^ cells/mL)	4	1.25–7 y	In all patients, good integration of the implants with host bone; no late fractures in the implant zone

HA: hydroxyapatite, TCP: tricalcium phosphate, and BMSCs: bone marrow derived mesenchymal stem cell.
